# Impact of improved observed hand hygiene on bloodstream infection rates in Ireland. A prospective segmented regression analysis, 2009–2016

**DOI:** 10.1017/S095026882000076X

**Published:** 2020-04-02

**Authors:** M.P. Smiddy, O.M. Murphy, E. Savage, A.P. Fitzgerald, S. FitzGerald, J. Browne

**Affiliations:** 1 School of Public Health, University College Cork, Ireland; 2 Pathology Department, Bon Secours Hospital, Cork, Ireland; 3 Catherine McCauley School of Nursing and Midwifery, University College Cork, Ireland; 4 Department of Statistics, University College Cork, Ireland; 5 Microbiology Department, St Vincent's University Hospital, Dublin 4, Ireland

**Keywords:** Bloodstream infections, Enterococcus, Hand hygiene, *Staphylococcus aureus*, Surveillance

## Abstract

Participation in European surveillance for bloodstream infection (BSI) commenced in Ireland in 1999 with all laboratories (*n* = 39) participating by 2014. Observational hand hygiene auditing (OHHA) was implemented in 2011. The aim of this study was to evaluate the impact of OHHA on hand hygiene compliance, alcohol hand rub (AHR) procurement and the incidence of sensitive and resistant *Staphylococcus aureus* and *Enterococcus faecium* and *faecalis* BSI. A prospective segmented regression analysis was performed to determine the temporal association between OHHA and outcomes. Observed hand hygiene improved from 74.7% (73.7–75.6) in 2011 to 90.8% (90.1–91.3) in 2016. AHR procurement increased from 20.1 l/1000 bed days used (BDU) in 2009 to 33.2 l/1000 BDU in 2016. A pre-intervention reduction of 2% per quarter in the ratio of methicillin sensitive *Staphylococcus aureus* BSI/BDU stabilized in the time period after the intervention (*P* < 0.01). The ratio of Methicillin resistant *Staphylococcus aureus* (MRSA) BSI/BDU was decreasing by 5% per quarter pre-intervention, this slowed to 2% per quarter post intervention, (*P* < 0.01). There was no significant change in the ratio of vancomycin sensitive (*P* = 0.49) or vancomycin resistant (*P* = 0.90) *Enterococcus* sp. BSI/BDU post intervention. This study shows national OHHA increased observed hand hygiene compliance and AHR procurement, however there was no associated reduction in BSI.

## Introduction

Bloodstream infection (BSI) is a serious complication of healthcare with mortality estimated at 15.3% of cases (95% CI: 14.8–15.8) in Ireland [1]. Participation in European surveillance for BSI commenced in Ireland in 1999 [2]. National engagement with this surveillance was enthusiastic with complete coverage by 2007 and all laboratories (*n* = 37) continuing to participate in 2016 with an estimated overall population coverage of 99% [3]. The Health Service Executive (HSE) is the organization which provides structure and governance for public health services provision in Ireland. Public reporting of hospital specific BSI data commenced in 2008 on the recommendation of the Health Service Executive strategy for the prevention and control of healthcare-associated infection (HCAI), with the first report published in 2009 [4]. While *Staphylococcus aureus* and enterococci have different reservoirs they are common causes of hospital acquired BSI [5]. Antimicrobial resistant BSI is more difficult to treat and result in higher morbidity and mortality [6, 7]. In Europe between 2013 and 2016 the reported prevalence of methicillin resistant *Staphylococcus aureus* (MRSA) BSI reduced significantly while there was no significant change in the overall prevalence of vancomycin resistant enterococci (VRE) BSI [8].

HCAIs cause significant morbidity and mortality worldwide [9]. Compliance with recommended hand hygiene guidelines reduces the risk of acquiring HCAI and improves patient safety [10]. The World Health Organization (WHO) recommends implementation of a multimodal intervention strategy to improve hand hygiene compliance [11]. This strategy includes training and education, observation of hand hygiene practice with feedback, provision of hand hygiene reminders, system change with provision of adequate resources and creation of an organizational safety culture. Observational hand hygiene auditing (OHHA) involves a trained observer monitoring and recording healthcare worker compliance with the WHO ‘five moments for hand hygiene’ [12]. Compliance is calculated as a percentage using the total number of opportunities available for hand hygiene as the denominator and the opportunities taken by the healthcare worker as the numerator. OHHA using a standardized methodology was implemented initially in the HSE in 2011. Each hospital site was required to report between 180 and 210 observations per biannual audit. These data were collected from a maximum of seven units per hospital with 44 hospitals participating in 2016. The units to be audited were selected by a computer programme reducing the risk of selection bias. Observed hand hygiene compliance was 74.7% (73.7–75.6) in 2011 and increased to 90.8% (90.1–91.3) in 2016 [13].

In this study we describe the impact of OHHA on the rate of hand hygiene compliance, alcohol hand rub (AHR) procurement and the prevalence of methicillin sensitive *Staphylococcus aureus* (MSSA), MRSA, vancomycin sensitive enterococci (VSE) and VRE BSIs in Irish hospitals over an 8-year period. BSIs were chosen as the outcome measure as there was no other suitable robust national infection data set available to assist in the evaluation of the hand hygiene audit intervention for the time period in question.

## Methods

### Study design

A prospective longitudinal study design comparing trends before and after the OHHA intervention. This design is recommended when randomized controlled trials are not feasible or unethical [14].

### Population

The research intervention sites included all acute hospitals in Ireland, with approximately 14 000 acute HSE beds in 2007 [15]. These sites catered for an estimated catchment population of 4 761 865 in 2016.

### Intervention

Nationally reported biannual OHHA which commenced in March/April 2011 [13].

### Control

The intervention was implemented both in quarter one and two in 2011 dependent on the hospital site. For the purpose of the segmented regression analysis the pre-intervention period extends from quarter one 2009 to quarter four 2010. This period acts as a control for the analysis. An intervention gap to allow for the effect of the OHHA includes quarter one and two of 2011. The post intervention period extends from quarter three 2011 quarter four 2016.

### Outcome

The primary outcome was data from the European Antimicrobial Resistance Surveillance Network (EARS-Net). The aim of EARS-Net is to create comparable, accurate antimicrobial resistance data that represents trends in Europe to inform policy decisions and stewardship interventions. EARS-Net collects clinical antimicrobial susceptibility testing data on the first isolate per patient per quarter of eight different pathogens [16]. BSI was defined using the Centers of Disease Control and Prevention/National Healthcare Safety Network definitions [17]. For the purpose of this study MSSA, MRSA, VSE and VRE expressed as the ratio of BSI cases per 1000 bed days used (BDU) (range 940 703–1 013 086) were analysed [18]. Secondary outcomes were the rate of observed hand hygiene compliance and the volume of AHR (litres/1000 BDU) procured.

### Statistical analysis

We calculated the rates of hand hygiene compliance and AHR and, ratios of BSI to BDU. A segmented regression analysis was carried out using a generalized linear model with a log link and robust variance.
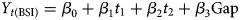
where,

*Y_t (_*_BSI*) =*_ *ln* (mean number of BSI cases per 1000 BDU)

*t*_1_ *=* *t* *−* 11 *for t* ⩽ 11, 0 *for t* *>* 11

*t*_2_ *=* *t* *−* 11 *for t* ⩾ 11, 0 *for t* *<* 11

Gap *=* 1 *if t* *=* 9 *or t* *=* 10, 0 *for t* *<* 9 *or t* *>* 10 [19]

An intervention gap from 1st January 2011 to 30th June 2011 was used in the interrupted analysis to allow for the bedding in of the intervention. We fitted models that looked for a change in level and change in slope. The slopes before and after intervention were compared using a Wald test. Results are presented as estimates with 95% confidence intervals. Statistical significance was defined as *P* < 0.05. Analyses were performed using Stata version 14 [20].

### Ethical approval

Ethical approval for the study was granted by the relevant ethics committee.

## Results

Observed hand hygiene compliance increased significantly from 74.6% (73.7–75.6) in the first audit in 2011 to 90.8% (90.1–91.3) in audit 12 in 2016 (*P* < 0.001). The hand hygiene outcome variables are presented per year in Table 1.

**Table 1.** Data on hand hygiene audits by year and audit number, 2011–2016

	Year											
	2011		2012		2013		2014		2015		2016	
Audit number	1	2	3	4	5	6	7	8	9	10	11	12
No. of sites	36	42	43	42	42	42	44	44	44	44	44	44
HSE HH target (%)	75	75	85	85	90	90	90	90	90	90	90	90
Opportunities for hand hygiene	7515	8765	8967	8779	8800	8567	9214	9216	9203	9210	9199	9228
Opportunities taken	5610	6975	7316	7399	7946	7391	7886	8032	8095	8213	8304	8375
Reported OHHA compliance (%)	74.7	79.6	81.6	84.3	85.2	86.2	85.6	87.2	88	89.2	90.3	90.8

**Fig. 1.**
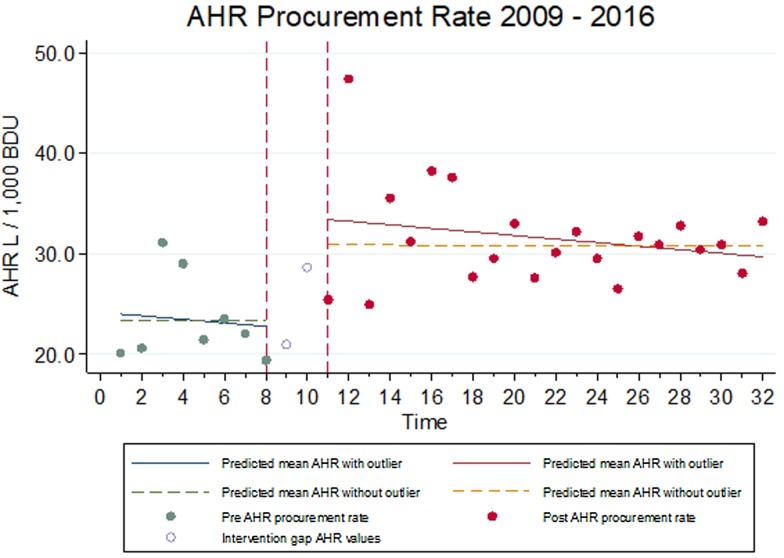
Alcohol hand rub procurement rates pre and post-OHHA intervention. *X* axis: *t* = 0 = Quarter 1 2009, *t* = 8 = Quarter 4 2010, *t* = 11 = Quarter 3 2011, *t* = 32 = Quarter 4 2016.

The rate of national AHR procurement increased from 20.1 l/1000 BDU in quarter one, 2009 to 33.2 l/1000 BDU in quarter four, 2016. Due to the presence of an outlier in quarter three 2011, models that included and excluded the outlier were run. Whether this outlier was included or not, there was no significant difference in slope before and after the OHHA intervention (*P* = 0.76). However, there was a significant jump with an increase following the intervention, (*P* < 0.01), Figure 1.

BSIs were tested for a change in slope both pre and post-intervention, Table 2.

**Table 2.** Interrupted time series of BSI outcome variables

	Slope before (quarterly rate of decline in variable before OHHA intervention) with 95% confidence interval	Slope after (quarterly rate of decline trend in variable after OHHA intervention) with 95% confidence interval	*P* value for change in slope.
MSSA BSI	0.98 (0.97–1.01)	1.00 (1.00–1.00)	*P* = 0.008
MRSA BSI	0.95 (0.93–0.97)	0.98 (0.97–0.99)	*P* = 0.007
VSE BSI	0.99 (0.96–1.01)	1.01 (1.01–1.01)	*P* = 0.49
VRE BSI	1.01 (0.96–1.01)	1.01 (1.00–1.02)	*P* = 0.90

The ratio of MSSA BSI/BDU was decreasing by 2% per quarter prior to the intervention. After the introduction of OHHA, the decline levelled off with 0.01% change per quarter. There is a significant difference when the ratios from the pre and post-intervention periods were compared (*P* < 0.01).

**Fig. 2.**
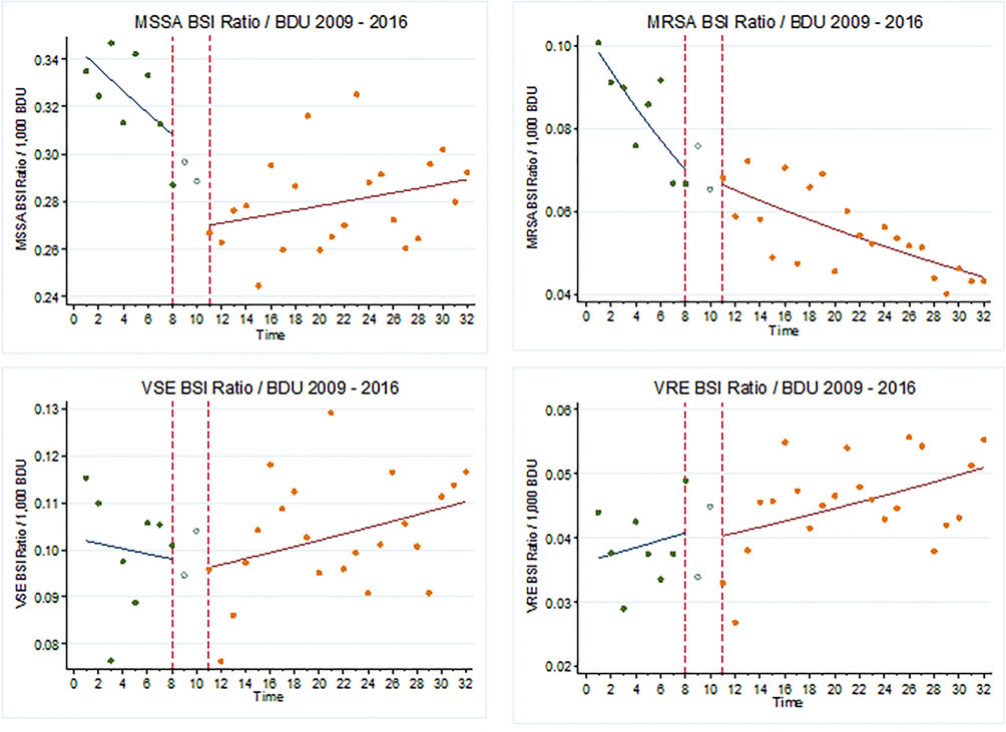
Ratio of BSI/BDU per Quarter 2009–2016 *X* axis: *t* = 0 = Quarter 1 2009, *t* = 8 = Quarter 4 2010, *t* = 11 = Quarter 3 2011, *t* = 32 = Quarter 4 2016.

The ratio of MRSA BSI/BDU was decreasing by 5% per quarter pre-intervention and continued to decrease by 2% per quarter post-intervention with a significant change (*P* < 0.01), Figure 2, Table 2.

The ratio of VSE BSI/BDU decreased by 1% per quarter pre-intervention; however, increased by 1% in the post-intervention period, but the change was not significant, (*P* = 0.49). The ratio of VRE BSI/BDU remained stable pre and post-intervention with no significant difference (*P* = 0.90), Table 2, Figure 2.

## Discussion

Observed hand hygiene compliance improved over the study period. This is consistent with other studies where OHHA was implemented [21, 22]. There was also a significant increase, which was maintained after the intervention, in the procurement of AHR which acts as a proxy for increased hand hygiene [23]. There was one outlier data point for AHR procurement reported in quarter four 2011. This could be due to a reporting anomaly or increased procurement could have been an initial behavioural change response to the OHHA intervention [24]. The data were analysed without the outlier data point in order to minimize the effect of the reported level of procurement which was not maintained over the study period.

The observed improvement in hand hygiene practices did not have a positive impact on the prevalence of BSIs. In this study the ratio of both MSSA and MRSA per BDU was falling prior to the introduction of the intervention. This is consistent with the reported European trends [8]. The reduction in MSSA levelled off after the intervention with no further reduction, consistent with previous studies [25, 26]. While the ratio of MRSA per BDU continued to reduce after the introduction of OHHA, the rate of fall slowed. As the reduction in both sensitive and resistant *Staphylococcus aureus* BSI was evident prior to the intervention, other factors such as antimicrobial resistance policy implementation [27], launch of prevention of intravascular catheter-related guidelines [28] and national publication of hospital BSI rates [4] may also have influenced this improvement. Open disclosure of hospital specific BSI rates was a major step towards transparency and the first time such clinical data were made publically available in Ireland. Varied hand hygiene and infection prevention and control interventions have demonstrated efficacy in reducing the incidence of MRSA [25, 26, 29]. The lack of intervention effect may also be partially attributable to the source of acquisition of BSI, with an estimated 48% of MSSA BSI infections acquired in the community setting [3]. However, there is some evidence to suggest that the reductions in *Staphylococcus aureus* BSI are related to incompletely understood biological changes in the organism [30].

The ratio of VSE BSI per BDU was increasing pre-intervention and continued to increase. The ratio of VRE BSI per BDU remained unchanged in 2016. Ireland has one of the highest proportions of VRE BSI in Europe and continues to increase across the continent [8]. While environmental contamination with both *Staphylococcus aureus* and *Enterococcus* spp. are possible sources of infection, enterococci are more prevalent environmental pathogens [31, 32]. VRE can survive severe conditions withstanding cleaning and disinfection to survive potentially years in the environment [33]. The environmental reservoir has been indicated as a source of spread of VRE colonisation and infection [34]. While hand hygiene is a critical component in preventing HCAI [35] rigorous environmental hygiene is essential to reduce the risk of transmission of *Enterococcus* spp. [36]. In addition to environmental hygiene strict isolation precautions, screening and antimicrobial stewardship are essential to control the spread of VRE [37].

Overall there was no association between a reduction in any of the BSI outcome variables and the OHHA intervention. However, MRSA BSI/BDU ratios continued to fall and while not significant, OHHA may have been a contributing factor. The lack of an association may be due to the complexity of factors that may influence BSI of which hand hygiene is just one element.

The strengths of this study include the use of nationally reported datasets where data have been reported consistently over time and the addition of AHR procurement data as an additional proxy measure for hand hygiene. However, the AHR data reflects procurement only and there is no way to discriminate between healthcare worker and patient/visitor usage. Limitations include the lack of more observed hand hygiene data points and the inability to differentiate between healthcare associated BSI and community acquired BSI using the national data set. This second point could contribute to the lack of effect on BSI incidence as the hand hygiene intervention was aimed at reducing healthcare associated infections. In addition, other infection prevention and control measures that occurred during the study period could have influenced the outcome measures. These include the launch and implementation of guidelines for antimicrobial stewardship in hospitals in Ireland [27] and guidelines on the prevention of intravascular catheter-related infection [28] in 2009.

## Conclusions

Implementation of national OHHA has resulted in a significant and maintained improvement in observed hand hygiene compliance and AHR procurement. OHHA positively influences hand hygiene behaviours, however, this alone does not translate to improvement in BSI outcomes.
